# Supplementary material for: Unraveling the origin of Kondo-like behavior in the 3d-electron heavy-fermion compound YFe2Ge2
Source: Proc Natl Acad Sci U S A. 2024 Sep 19;121(39):e2401430121. doi: 10.1073/pnas.2401430121 (PMC11441551; doi:10.1073/pnas.2401430121)
Supplement: Supplementary file 1 — Appendix 01 (PDF) [file pnas.2401430121.sapp.pdf]

PNAS
www.pnas.org

Supplementary Information for

Unraveling the origin of Kondo-like behavior in the 3d-electron heavy-fermion compound YFe_2Ge_2

Bing Xu, et al.

Bing Xu, Zhiping Yin, Christian Bernhard.
E-mail: bingxu@iphy.ac.cn, yinzhiping@bnu.edu.cn, christian.bernhard@unifr.ch

This PDF file includes:

Supplementary text
Figs. S1 to S12 (not allowed for Brief Reports)
Table S1 (not allowed for Brief Reports)
SI References

Supporting Information Text

A: Experimental details

High-quality single crystals of YFe_2Ge_2 were grown with a Sn-flux method (1). The resistivity versus temperature was measured with a commercial Physical Properties Measurement System (Quantum Design PPMS), as shown in the inset of Fig. 3A in the main text. A crystal with a mirror-like ab surface with a size of about 3 × 3 mm was used for the optical measurements. The ab-plane reflectivity $R(\omega)$ was measured at near-normal incidence with a Bruker VERTEX 70V Fourier transform infrared spectrometer. An *in situ* gold overfilling technique (2) was used to obtain the absolute reflectivity. The reflectivity spectra have been achieved over a broad frequency range from approximately 4 meV to 2 eV by using a series of combinations of sources, beamsplitters, and detectors. The reflectivity spectra at different temperatures from 300 K to 10 K were collected with an ARS-Helitran cryostat.

The optical response functions, like the complex dielectric function $\tilde{\varepsilon}(\omega) = \varepsilon_1(\omega)+i\varepsilon_2(\omega)$ and the complex optical conductivity $\tilde{\sigma}(\omega) = \sigma_1(\omega) + i\sigma_2(\omega) = -2\pi i\omega[\tilde{\varepsilon}(\omega) - \varepsilon_\infty]/Z_0$ (in units of $\Omega^{-1}\text{cm}^{-1}$, where $Z_0 = 377\ \Omega$ is the vacuum impedance), were obtained from a Kramers-Kronig analysis of $R(\omega)$ (3). For the low-frequency extrapolation, we used the function $R(\omega) = 1-A\sqrt{\omega}$ (Hagen-Rubens). On the high-frequency side, we used the ellipsometry data, for which the spectrum in the near-infrared to ultraviolet range (0.5 – 6 eV) was measured at room temperature with a commercial ellipsometer (Woollam VASE). For frequencies above 6 eV, we used a constant reflectivity up to 12.5 eV followed by a free-electron (ω^{-4}) termination. Figure S1 confirms that the spectrum derived from the Kramers-Kronig analysis matches quite well with the ellipsometry data in the range from 0.5 – 6 eV.

B: Drude-Lorentz model analysis

For a quantitative analysis of the optical data, we fitted the measured reflectivity $R(\omega)$ and the complex optical conductivity $\tilde{\sigma}(\omega) = \sigma_1(\omega) + i\sigma_2(\omega)$ using a Drude-Lorentz model. In terms of the complex dielectric function this model can be written as:

$$\tilde{\varepsilon}(\omega) = \varepsilon_\infty + \sum_j \frac{\omega_{pj}^2}{\omega_{0j}^2 - \omega^2 - i\gamma_j\omega}. \qquad [1]$$

It describes the optical response of a set of harmonic oscillators, each independently characterized by three parameters: a resonance frequency ω_{0j}, a linewidth γ_j, and a plasma frequency ω_{pj} for the j-th Lorentz oscillator. For a Drude response, the resonance frequency ω_{0j} is set to zero. Here, ϵ_∞ is the high-frequency dielectric constant. The complex conductivity is related to the dielectric function by $\tilde{\sigma}(\omega) = \sigma_1(\omega) + i\sigma_2(\omega) = -2\pi i\omega[\tilde{\varepsilon}(\omega) - \varepsilon_\infty]/Z_0$. The normal-incidence reflectivity R is given by $R =\mid \frac{1-\sqrt{\tilde{\varepsilon}}}{1+\sqrt{\tilde{\varepsilon}}} \mid^2$.

Figures S2(A–C) show the measured $\sigma_1(\omega)$ spectra (black curves) of YFe_2Ge_2, and the corresponding fits (red curves) to the spectra at 300 K, 100 K and 10 K, respectively. The fitting curve is decomposed into the contributions of the coherent Drude response (D1), the incoherent Drude response (D2), and the α, and β bands. An additional small Lorentz term accounts for the M band that develops at low temperatures. Figures S2(D–F) show the corresponding fits to the $\sigma_2(\omega)$ spectra. The insets of Figures S2(D–F) display the corresponding fits to the reflectivity spectra.

C: Extended Drude model analysis

In the framework of the extended Drude model the complex optical conductivity is expressed as (4, 5):

$$\sigma(\omega) = \frac{2\pi}{Z_0}\frac{\Omega_p^2}{\tau^{-1}(\omega) - i\omega[1+\lambda(\omega)]}, \qquad [2]$$

where Ω_p and $Z_0 = 377\ \Omega$ are the plasma frequency and the vacuum impedance, respectively.

Eq. 2 contains a frequency-dependent scattering rate $1/\tau(\omega)$ and a mass enhancement factor $m^*(\omega)/m_b \equiv 1 + \lambda(\omega)$ that can be derived from the real $[\sigma_1(\omega)]$ and imaginary $[\sigma_2(\omega)]$ parts of the complex optical conductivity as follows:

$$\frac{1}{\tau(\omega)} = \frac{2\pi}{Z_0}\Omega_p^2\frac{\sigma_1(\omega)}{\sigma_1^2(\omega) + \sigma_2^2(\omega)}, \qquad [3]$$

$$\frac{m^*(\omega)}{m_b} = \frac{1}{\omega}\frac{2\pi}{Z_0}\Omega_p^2\frac{\sigma_2(\omega)}{\sigma_1^2(\omega) + \sigma_2^2(\omega)}. \qquad [4]$$

To extract $1/\tau(\omega)$ and $m^*(\omega)/m_b$ for the itinerant carriers one has to first subtract the interband contributions from both the real and imaginary part of the optical conductivity (6). Figure S3(A) displays the $\sigma_1(\omega)$ and $\sigma_2(\omega)$ spectra at 10 K (solid lines) and the corresponding interband contributions (dashed lines). Here, the interband contribution has been derived from the Drude-Lorentz model analysis. The temperature-dependent $\sigma_1(\omega)$ and $\sigma_2(\omega)$ spectra after this subtraction of the interband transitions are displayed in Fig. S3(B) and Fig. S3(C), respectively.

Finally, the plasma frequency Ω_p is obtained according to:

$$\Omega_p^2 = \frac{Z_0}{\pi^2}\int_0^\infty [\sigma_1^{\text{exp}}(\omega) - \sigma_1^{\text{inter}}(\omega)]d\omega, \qquad [5]$$

where $\sigma_1^{\exp}(\omega)$ and $\sigma_1^{\text{inter}}(\omega)$ represent the real part of the optical conductivity from experiment and the interband transition contribution, respectively. Figure S3(D) displays the temperature-dependent plasma frequency obtained with Eq. 5.

Subsequently, we utilize Eq. 3 and Eq. 4 to determine $1/\tau(\omega)$ and $m^*(\omega)/m_b$ for the itinerant carriers. Figure S3(E) and Figure S3(F) show the temperature evolution of the frequency-dependent scattering rate $1/\tau(\omega)$ and the mass enhancement $m^*(\omega)/m_b$, respectively. The insets show a comparison of $1/\tau(\omega)$ and $m^*(\omega)/m_b$ as obtained with and without the interband contributions. They confirm that the interband contribution does not strongly influence the low-energy part of the $1/\tau(\omega)$ and $m^*(\omega)/m_b$ spectra.

D: Computational details of electronic band structure calculations

The electronic band structure calculations on the static mean-field level are implemented using the all-electron code WIEN2k (7) that is built on the full-potential linear augmented plane wave method. The Perdew-Burke-Ernzerhof version (8) of the generalized gradient approximation is used as the exchange-correlation functional. The combination of density functional theory and dynamical mean field theory (DFT+DMFT) (9, 10) is employed to calculate the many-body electronic structure. For both DFT and DFT+DMFT charge self-consistent calculations, a 21 × 21 × 21 k-point grid is used. In the calculations of the optical conductivity, a denser 46 × 46 × 46 k-point grid is adopted. The muffin-tin radii are 2.50, 2.38, and 2.11 Bohr radii for Y, Fe, and Ge, respectively, and $R_{\text{mt}} \times K_{\text{max}}$ is 8.0. The quantum impurity problem has been solved by the continuous time quantum Monte Carlo (CTQMC) method (11, 12). All of the five Fe-3d orbitals are considered as correlated ones and the fully rotational invariant form is applied for a local Coulomb interaction Hamiltonian with an on-site Coulomb repulsion $U = 5.0$ eV and a Hund's coupling $J_H = 0.8$ eV, which is consistent with previous DFT+DMFT calculations of iron-based superconductors (13, 14).

The main results of the DFT+DMFT are summarized in Figure 5 in the main text. For a direct comparison, Figure S4 shows the band structure of YFe_2Ge_2 as obtained using the DFT+DMFT method at $T = 290$ K and the DFT method (yellow lines). Note that the imaginary part of the self-energy has been intentionally set to a ten times smaller value as to enhance the sharpness of the bands in the DFT+DMFT calculations at $T = 290$ K, where the flat band and the hybridization gap persist at the Fermi level. Figure S5 shows the details of the temperature evolution of band structure and the DOS around the flat band and hybridization gap.

We have performed calculations to determine the orbital-resolved Fermi surfaces of YFe_2Ge_2 within the $k_z = 0$ plane at $T = 40$ K. As show in Fig. S6, the flat bands are located in regions surrounding the M(0.5, 0.5) point, maintaining a distance from the Fermi level smaller than the width of the band broadening. This results in the flat bands to retain a significant weight at the Fermi level, which is visually represented in Fig. S6 as sizable regions of color. In contrast, the two circular Fermi surfaces near the Z′(1.0, 0.0) point are associated with bands that exhibit more pronounced dispersion, resulting in more confined cross-sections of the Fermi surface. Utilizing half the height of the spectral function peak at the M point as a benchmark for the flat bands being at the Fermi level, we have estimated that 15.6% of the first Brillouin zone within the $k_z = 0$ plane contribute to the flat band.

The orbital-resolved mass enhancements are obtained by fitting a fourth-order polynomial through the lowest six points and the origin using the equation: $m^*/m_b = 1 - \lim_{\omega\to 0^+} \frac{\partial \Sigma(\mathrm{i}\omega)}{\partial \omega}$.

E: A comparison of the DFT+DMFT calculations

In this section, we added a comparative discussion between our DFT+DMFT calculations and those from a previous study by Skornyakov *et al.* (15) Unlike the previous study, we employed a different code to perform DFT+DMFT calculations with experimentally determined crystal structure (16), which is obtained through optimization in the previous study (15). We opted for a larger U value of 5 eV and a slightly smaller J_H value of 0.8 eV, while the previous study employed U and J_H values of 3 eV and 0.85 eV, respectively. To facilitate a comparison of the electronic structures, we computed the band structure along the high-symmetry paths used in the previous study. Overall, as shown in Fig. S7, the band structures presented in the previous study are consistent with ours. However, our parameter selections have led to a somewhat larger mass enhancement and a shift in the position of the flat bands. In the previous study, the flat bands are slightly lower (~ -0.05 eV) and do not fall at the Fermi level as they do in our calculations.

F: An estimation of the Sommerfeld coefficient

To estimate the thermodynamic properties, we calculated the DOS across the entire Brillouin zone at $T = 40$ K. Despite YFe_2Ge_2 showing increased coherence at 40 K, there remains a slight incoherent component compared to 0 K. This residual incoherence leads to a broadening of the DOS and results in smaller peak values. Therefore, we reduced the imaginary component of the self-energy, which accounts for the incoherent broadening, to obtain the DOS at the Fermi level. As shown in Fig. S7, the obtained value of the DOS at the Fermi level is as high as 24.3 states/eV, which corresponds to an electronic specific heat γ of 57.3 mJ mol^{-1} K^{-2}. Here, the contribution to the DOS at the Fermi level is predominantly attributable to the t_{2g} orbitals, and the mass enhancement for the t_{2g} orbitals at 40 K is averaged to approximately 5.7 when weighted by the DOS. Furthermore, extrapolating to 0 K with a mass enhancement of around 9, it would yield an estimated Sommerfeld coefficient γ of about 90 mJ mol^{-1} K^{-2}, which is close to the value of 100 mJ mol^{-1} K^{-2} reported in previous studies (17, 18).

G: The determinations of the crossover temperature T^*

In addition to the estimations of the crossover temperature T^* obtained in terms of dielectric function, scattering rate, and mass enhancement as described in the main text, in this section, we introduce two additional methods to estimate T^*. Firstly, we use the maximum of the first derivative of the resistivity to characterize the coherence-incoherence crossover temperature T^*. As depicted in Fig. S9, the crossover temperature for YFe_2Ge_2 is estimated to be $T \sim 75$ K. An additional approach to trace the crossover temperature T^* is to examine the temperature evolution of the spectral weight in a specific region of the infrared response. As shown in Fig. S9 we analyze the spectral weight in the range of 50 – 200 cm^{-1}. In this region, below the temperature T^* around 100 K, the spectral weight is suppressed both by the narrowing of the coherent Drude peak and the appearance of the M peak. Overall, considering various approaches, the temperature T^* appears to be around 100 ± 25 K.

H: A comparison between KFe_2As_2 and YFe_2Ge_2.

Figure S10 shows a direct comparison of the optical spectra of KFe_2As_2 and YFe_2Ge_2. It is evident that both compounds exhibit striking similarities in their low-energy responses, such as a sharp Drude peak, a coherence-incoherence crossover, and an enhanced optical effective mass. However, there is a notable difference between these two compounds in their high-energy responses. In KFe_2As_2, the high-energy optical conductivity is dominated by a single-peak feature with no strong spectral weight redistribution. In contrast, in YFe_2Ge_2, the high-energy optical conductivity is dominated by a double-peak feature which is subject to a strong spectral weight redistribution. As explained in the main text, these similarities and differences between KFe_2As_2 and YFe_2Ge_2 can be attributed to different mechanisms. In KFe_2As_2 it can be attributed to a Kondo-type scenario due to Hund's metal state with strongly orbital selective correlations, while in YFe_2Ge_2 the flat band and the related heavy fermion behavior arises primarily from a kinetic frustration and band hybridization effect, as described in the main part of the manuscript.

Figure S11 illustrates a direct comparison of the DFT band structures of KFe_2As_2 and YFe_2Ge_2. It demonstrates that the d bands in the vicinity of the Fermi level are considerably narrower in YFe_2Ge_2 than in KFe_2As_2. The narrowing of bands in YFe_2Ge_2 can be attributed to the so-called kinetic frustration effect (19). This arises due to a competition between the direct Fe-Fe and the indirect Fe-Ge-Fe hopping channels for which the hopping parameters have opposite signs and thus give rise to a destructive interference effect. In the case of YFe_2Ge_2, the collapsed 122-structure results in a stronger Fe-Ge bonding, enhancing the Fe-Ge-Fe hopping parameter compared to the uncollapsed 122-structure of KFe_2As_2. As a result, the comparable amplitudes of the direct and indirect hoppings give rise to a strong destructive interference effect in YFe_2Ge_2, thus leading to stronger band flattening. A detailed estimation of the hopping parameters for YFe_2Ge_2 is provided in the next section.

Another notable difference in the DFT band structures between KFe_2As_2 and YFe_2Ge_2 is the presence of band hybridization. In YFe_2Ge_2, the bands along both the $\Gamma - M$ and $\Gamma - X$ directions exhibit clear indications of band hybridization, whereas this feature is absent in KFe_2As_2. As depicted in Fig. S12, the orbital character analysis reveals that a portion of the Y $4d$ orbitals becomes involved with the Fe $3d$ orbitals near the Fermi level. This interaction arises from the partial occupation and the extended spatial expansion of the Y $4d$ electrons, which enable them to overlap with Fe $3d$ electrons. On the contrary, the s electrons of K in KFe_2As_2 act as electron donors, with their orbitals being completely empty and located at a higher energy level, hindering their hybridization with Fe $3d$ electrons.

I: An estimation of the Fe-Fe direct and indirect hopping parameters of YFe_2Ge_2.

To obtain the Fe-Fe direct and indirect hopping parameters, we construct maximally localized Wannier functions (MLWFs) (20) from the DFT band structure. The band structures constructed by MLWF tight binding model coincided well with that of the DFT calculations near the Fermi energy, including the Y($4d$), Fe($3d$), and Ge($4p$) orbitals. To clarify the hopping interactions between Fe atoms, we designate the two adjacent Fe atoms as Fe1 and Fe2. Since Fe2 is located on the y-axis relative to the local axis of Fe1, the direct hopping parameter for Fe1(d_{xz})–Fe2(d_{xz}) is negligible (0.006 eV), while that for Fe1(d_{yz})–Fe2(d_{yz}) is considerable (0.190 eV). The direct hopping parameter for Fe1(d_{xy})–Fe2(d_{xy}) is 0.205 eV. The indirect hopping of the Fe1(d_{xz})–Fe2(d_{xz}) or Fe1(d_{yz})–Fe2(d_{yz}) orbitals is primarily mediated through the Ge(p_x/p_y) orbitals. The hopping parameters of Fe1–Ge(p_x/p_y) and Fe2–Ge(p_x/p_y) have opposite signs, resulting in a negative product. As a result, the indirect hopping counteracts the direct hopping, leading to kinetic frustration of the d_{xz}/d_{yz} orbital. Similarly, the indirect hopping of the Fe1(d_{xy})–Fe2(d_{xy}) orbital is primarily mediated through the Ge(p_z) orbitals. The hopping parameters of Fe1–Ge(p_z) and Fe2–Ge(p_z) have opposite signs, leading to a negative product, which counteracts the direct hopping and results in kinetic frustration of the d_{xy} orbital. A summary of all hopping parameters is provided in Table S1.

Table S1. The DFT hopping parameters of the Fe(d_{xz},d_{xz},d_{xy}) orbitals to the neighboring Fe(d_{xz},d_{xz},d_{xy}) and Ge(p_x,p_y,p_z) orbitals obtained from the MLWFs in YFe_2Ge_2.

	Fe1–Fe2 (eV)	Fe1–Ge(p_x) (eV)	Fe2–Ge(p_x) (eV)	Fe1–Ge(p_y) (eV)	Fe2–Ge(p_y) (eV)	Fe1–Ge(p_z) (eV)	Fe2–Ge(p_z) (eV)
d_{xz}	0.006	−0.612	0.419	0.419	−0.612	−0.002	−0.002
d_{yz}	0.190	0.612	−0.419	0.419	−0.612	0.002	−0.002
d_{xy}	0.205	−0.277	0.000	0.000	0.277	0.613	−0.613

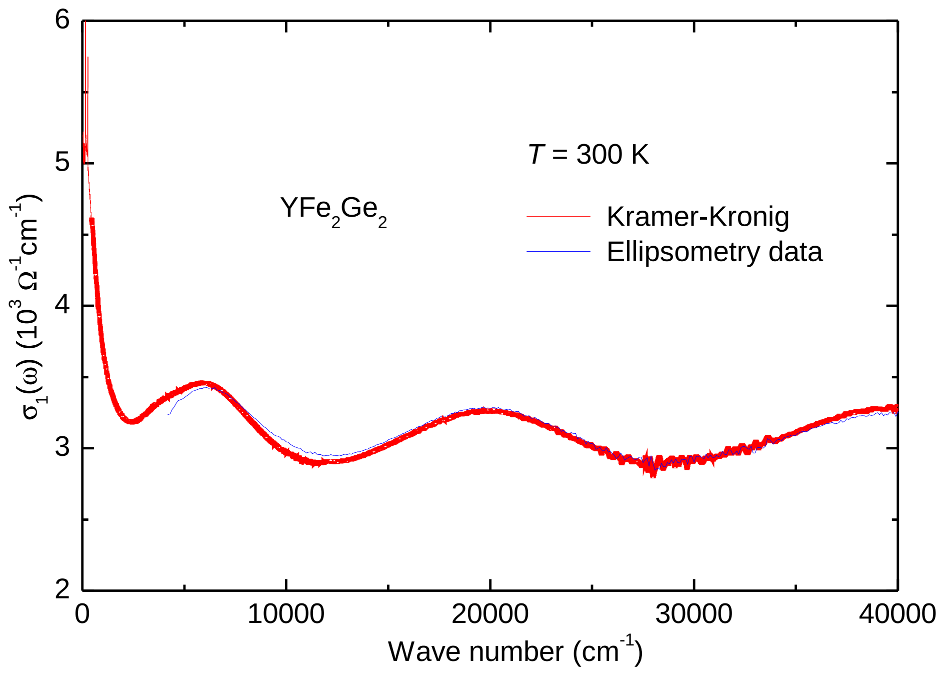

Fig. S1. (color online) Comparison of the $\sigma_1(\omega)$ spectrum of YFe_2Ge_2 at 300 K in the range up to 40 000 cm^{-1} as obtained from the ellipsometry data (blue line) and a Kramers-Kronig analysis of the reflectivity data (red line).

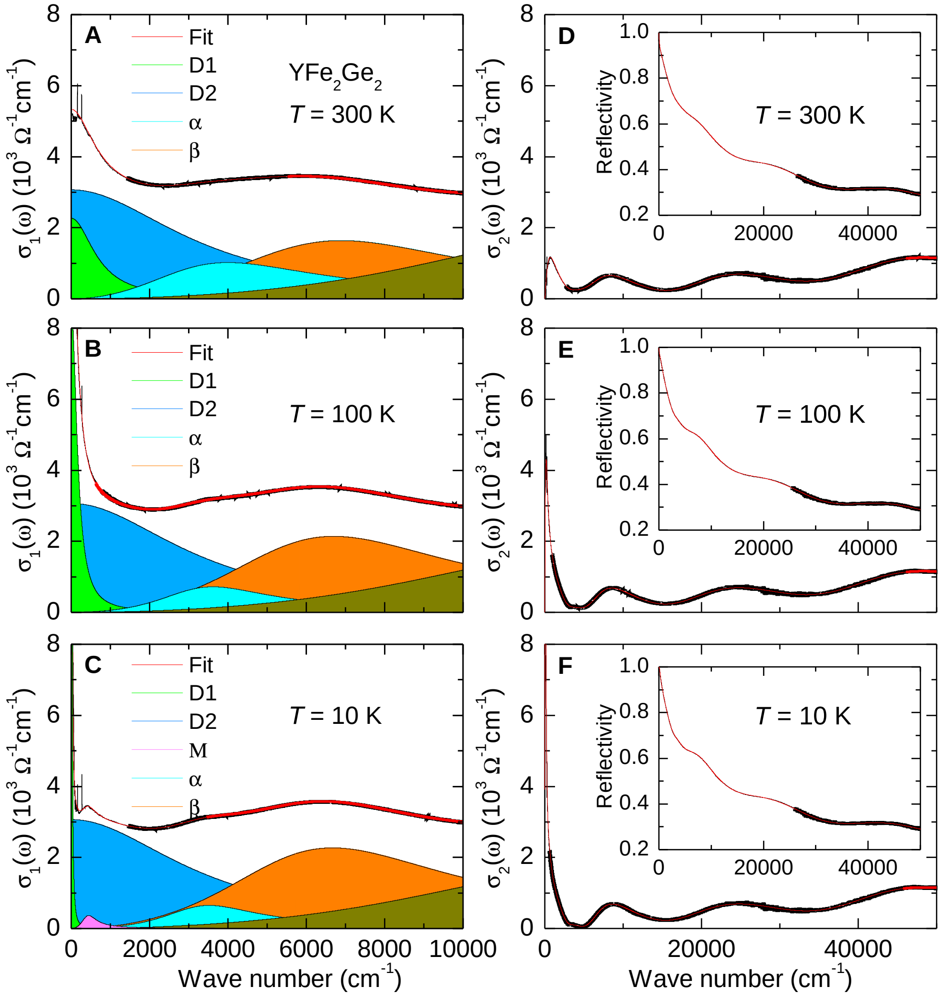

Fig. S2. (color online) (A–C) Decomposition of the $\sigma_1(\omega)$ spectra of YFe_2Ge_2 using a Drude-Lorentz model at $T = 300$ K, 100 K and 10 K, respectively. (D–F) Corresponding fits to the spectra of $\sigma_2(\omega)$. The inset shows the fits to the reflectivity spectra.

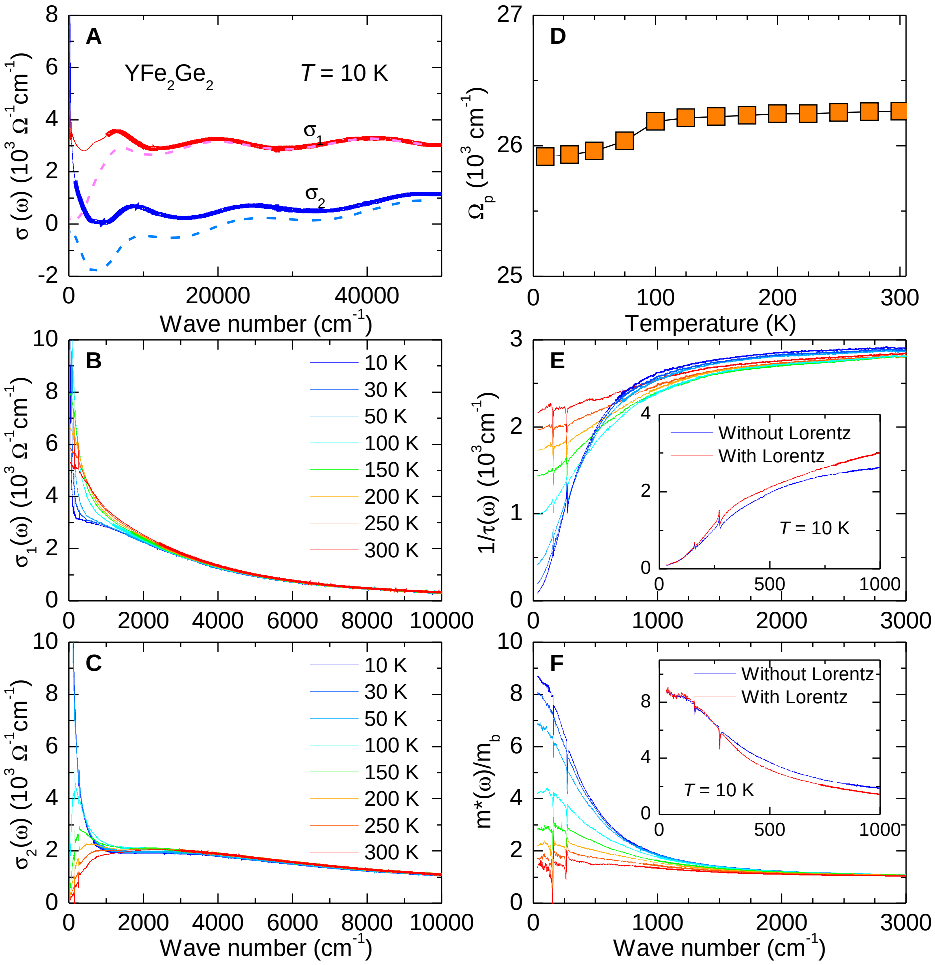

Fig. S3. (color online) (A) The $\sigma_1(\omega)$ and $\sigma_2(\omega)$ spectra at 10 K (solid lines) and the corresponding interband contributions (dashed lines). Temperature-dependent (B) $\sigma_1(\omega)$ and (C) $\sigma_2(\omega)$ spectra without the contributions of the interband excitations. (D) Temperature-dependent plasma frequency obtained from the spectral weight of the $\sigma_1(\omega)$ spectra without the interband contributions. (E) and (F) Temperature evolution of the frequency-dependent scattering rate $1/\tau(\omega)$ and mass enhancement $m^*(\omega)/m_b$, respectively, without the interband contributions. The corresponding insets show the frequency-dependent scattering rate $1/\tau(\omega)$ and mass enhancement $m^*(\omega)/m_b$ at 10 K with and without the interband contributions.

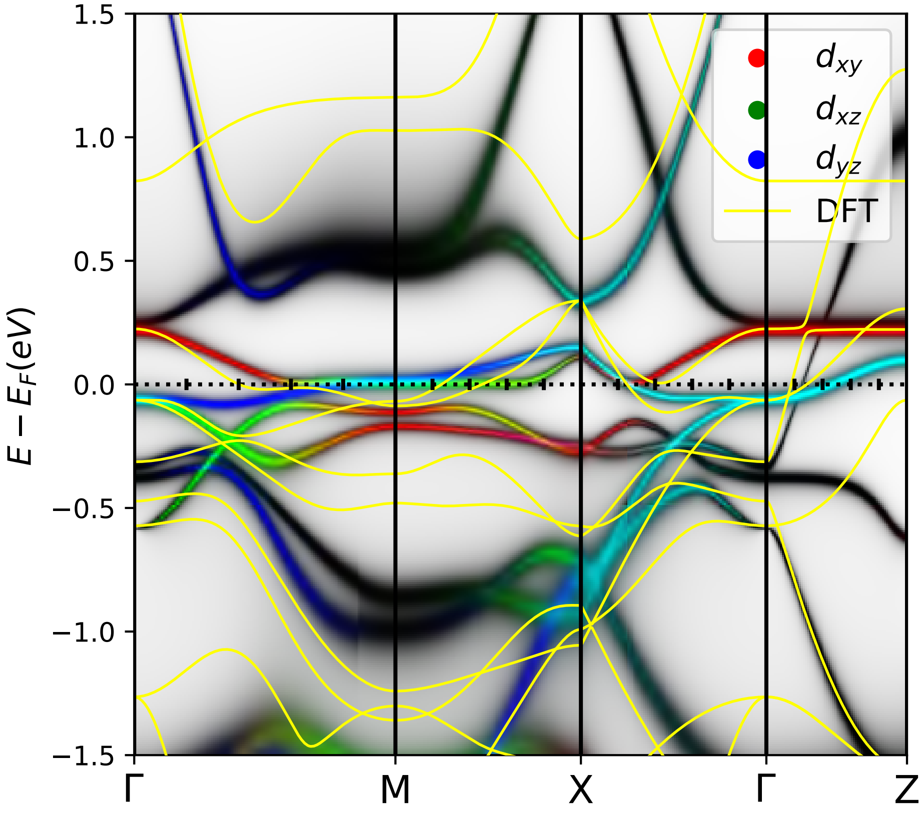

Fig. S4. (color online) A comparison of the electronic band structure of YFe_2Ge_2 obtained using the DFT+DMFT method at $T = 290$ K and the DFT method (yellow lines). Note that the imaginary part of the self-energy has been intentionally set ten times smaller to enhance the sharpness of the bands of the DFT+DMFT calculations at $T = 290$ K.

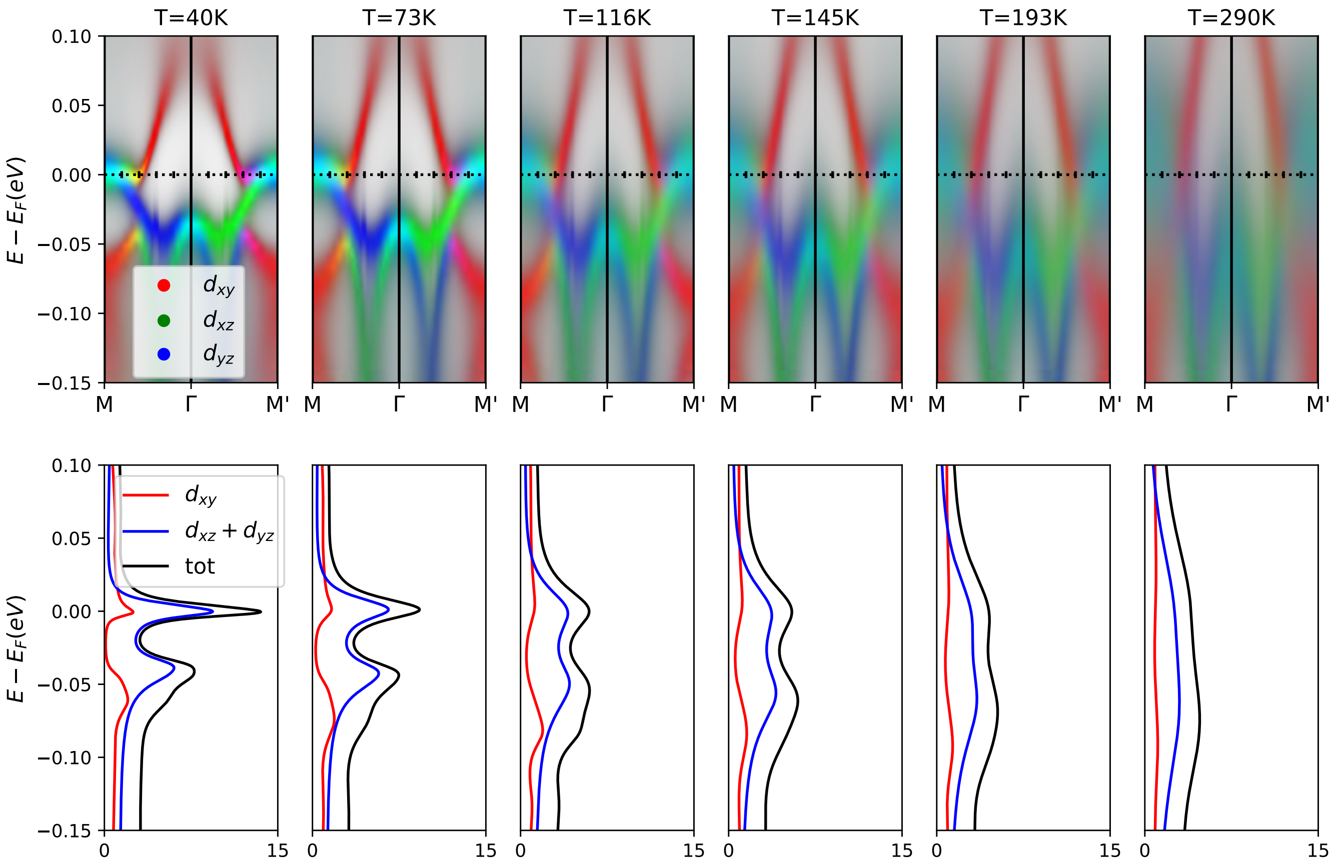

Fig. S5. (color online) Orbital-resolved electronic structure and DOS of YFe_2Ge_2 along the Γ–M/M$'$ directions near the Fermi level obtained using the DFT+DMFT method at different temperatures.

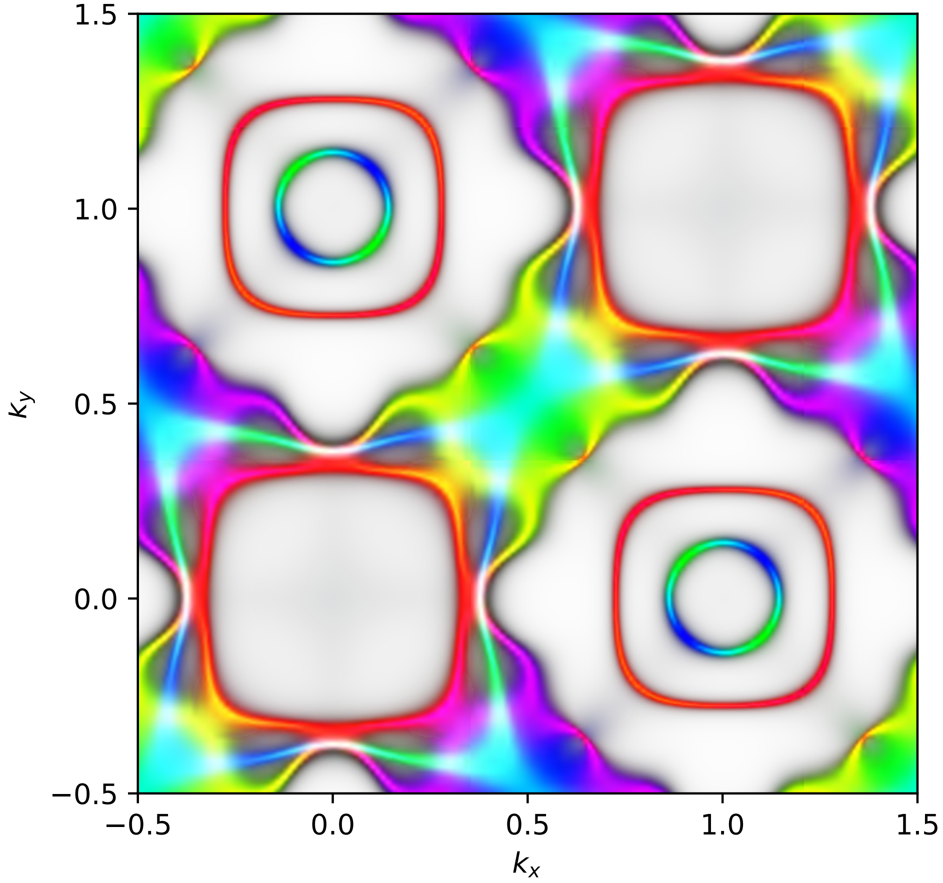

Fig. S6. (color online) Orbital-resolved Fermi surfaces of YFe_2Ge_2 within the kz =0 plane obtained using the DFT+DMFT method at $T = 40$ K.

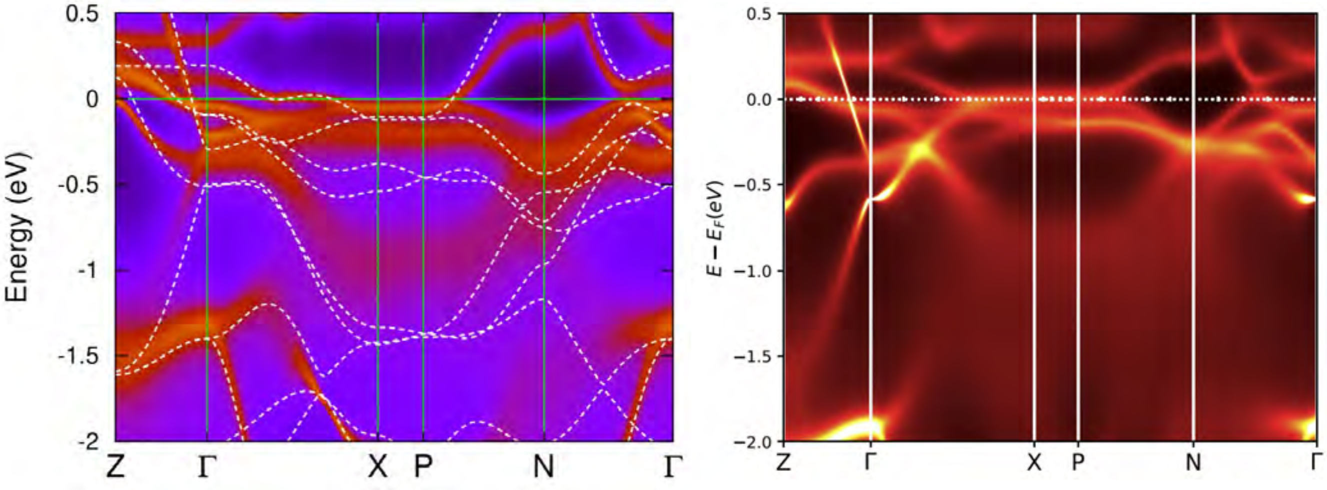

Fig. S7. (color online) Electronic structure of YFe_2Ge_2 obtained from DFT+DMFT calculations at $T = 290$ K. The left panel is the one of the previous study (15). The right panel is the one of our calculations.

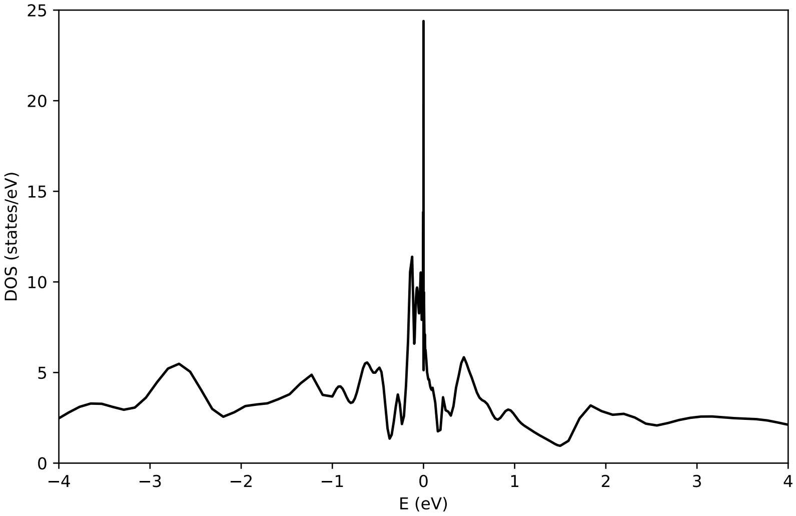

Fig. S8. (color online) Total DOS of YFe_2Ge_2 with DFT + DMFT calculations at $T = 40$ K.

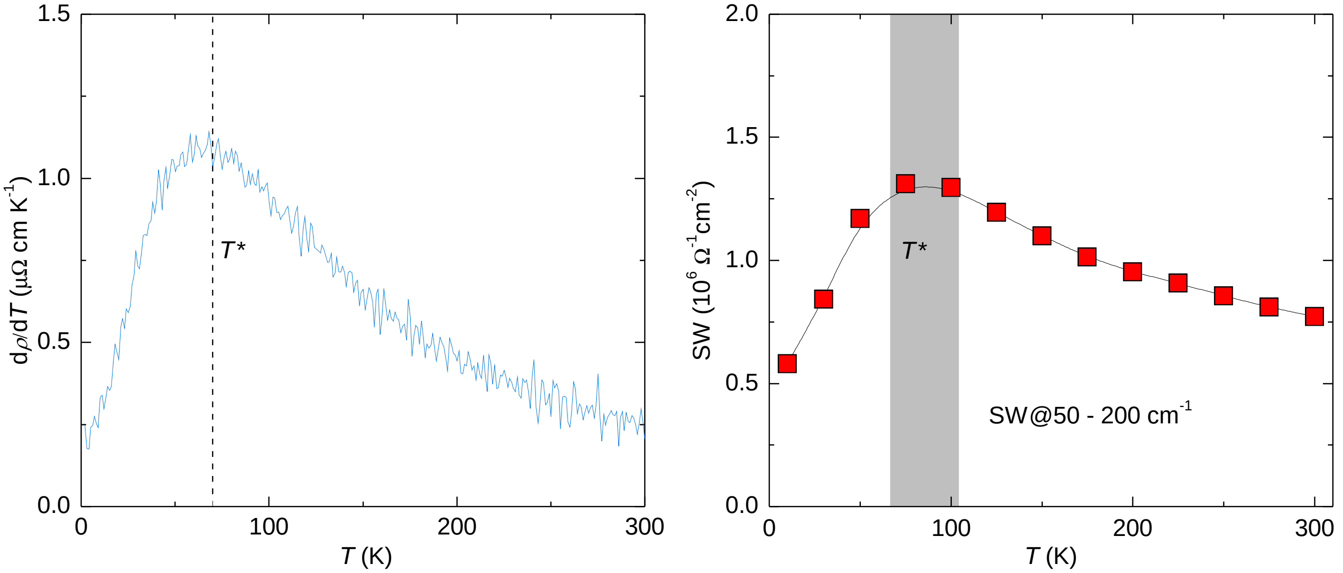

Fig. S9. (color online) Left panel shows the first derivative of the temperature-dependent resistivity of YFe_2G_2. Right panel shows the temperature dependence of the spectral weight changes ranged at 50 – 200 cm^{-1}.

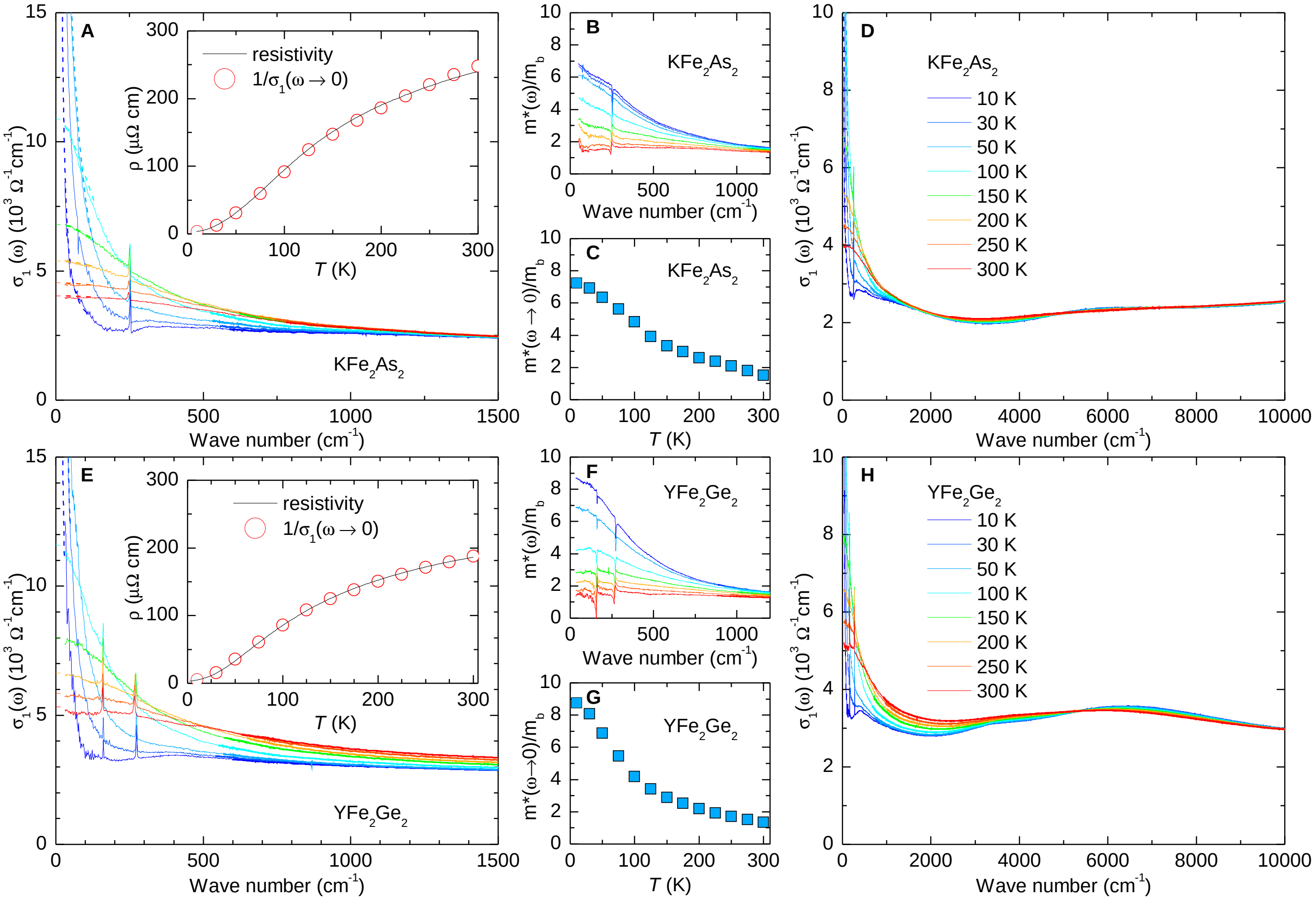

Fig. S10. (color online) Comparison of the optical response of KFe_2As_2 (upper panels) and YFe_2Ge_2 (lower panels). (A) Temperature-dependent spectra of the optical conductivity of KFe_2As_2. The inset shows a comparison with the dc resistivity. (B) and (C) Spectra of the frequency-dependent mass enhancement and their zero-frequency values as a function of temperature in KFe_2As_2. (D) High-energy part of the optical conductivity spectra of KFe_2As_2. (E–H) Similar plots as (A–D) for the case of YFe_2Ge_2.

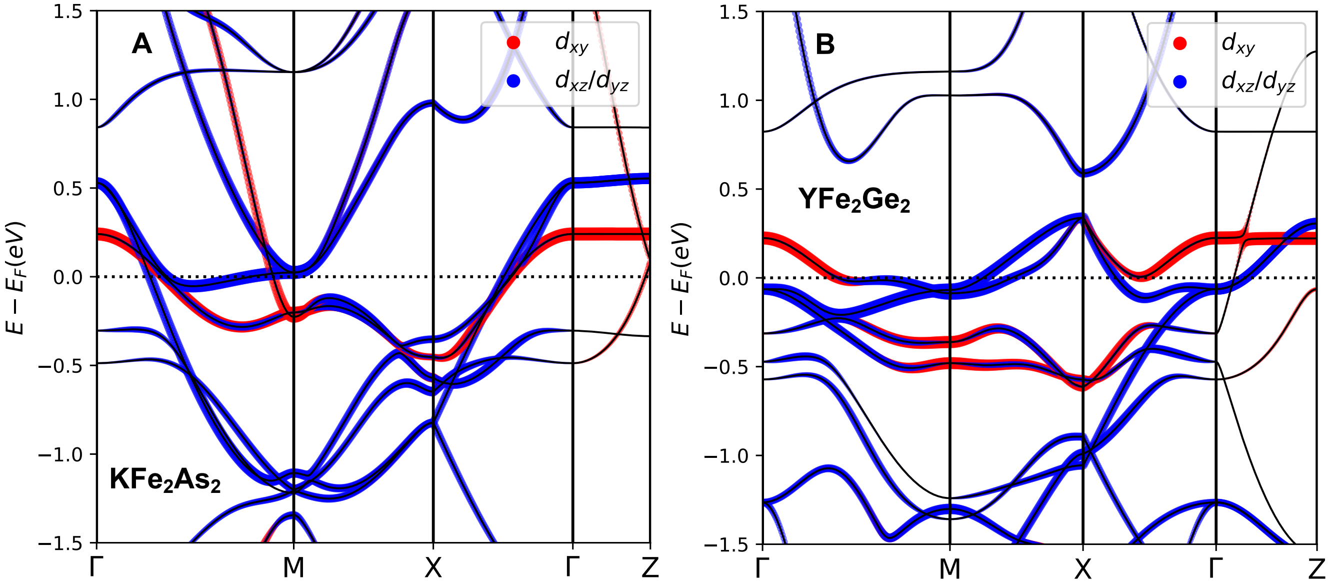

Fig. S11. (color online) Orbital-resolved electronic band structure for (A) KFe_2As_2 and (B) YFe_2Ge_2 calculated with the DFT method, which underestimates electronic correlations.

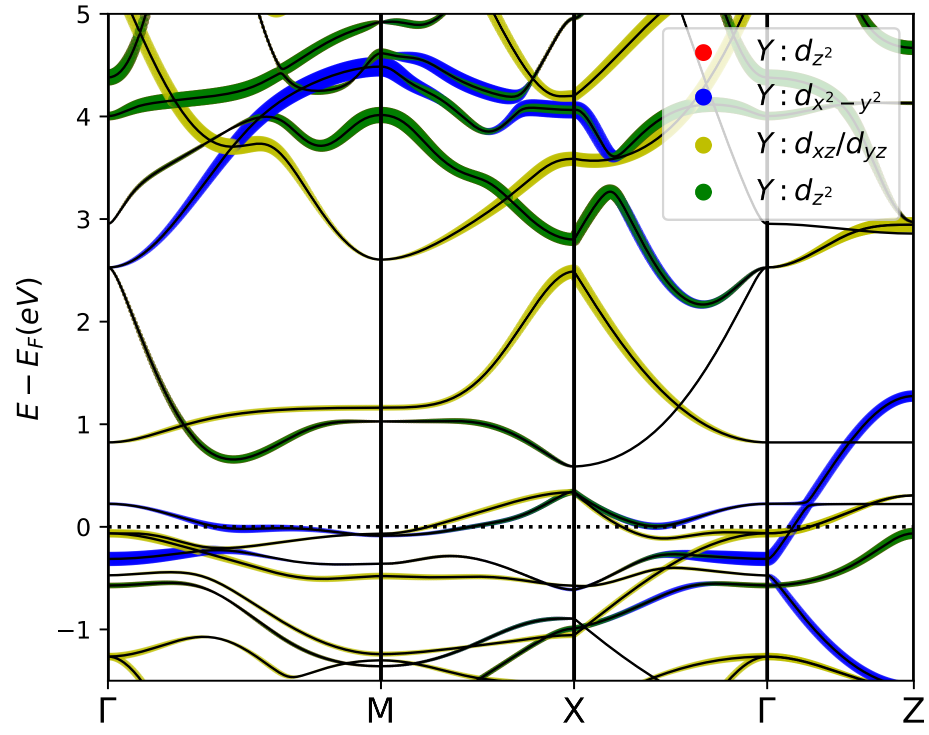

Fig. S12. (color online) Band structure of YFe_2Ge_2 calculated with the DFT method, showing the different orbital characters of Y $4d$ electrons.

References

1. H Wo, et al., Coexistence of Ferromagnetic and Stripe-Type Antiferromagnetic Spin Fluctuations in YFe_2Ge_2. *Phys. Rev. Lett.* **122**, 217003 (2019).
2. CC Homes, M Reedyk, DA Cradles, T Timusk, Technique for measuring the reflectance of irregular, submillimeter-sized samples. *Appl. Opt.* **32**, 2976–2983 (1993).
3. M Dressel, G Grüner, *Electrodynamics of Solids.* (Cambridge University press), (2002).
4. DN Basov, T Timusk, Electrodynamics of high-T_c superconductors. *Rev. Mod. Phys.* **77**, 721–779 (2005).
5. DN Basov, RD Averitt, D van der Marel, M Dressel, K Haule, Electrodynamics of correlated electron materials. *Rev. Mod. Phys.* **83**, 471–541 (2011).
6. L Benfatto, E Cappelluti, L Ortenzi, L Boeri, Extended drude model and role of interband transitions in the midinfrared spectra of pnictides. *Phys. Rev. B* **83**, 224514 (2011).
7. P Blaha, et al., WIEN2k: An APW+lo program for calculating the properties of solids. *The J. Chem. Phys.* **152**, 074101 (2020).
8. JP Perdew, K Burke, M Ernzerhof, Generalized Gradient Approximation Made Simple. *Phys. Rev. Lett.* **77**, 3865–3868 (1996).
9. G Kotliar, et al., Electronic structure calculations with dynamical mean-field theory. *Rev. Mod. Phys.* **78**, 865–951 (2006).
10. K Haule, CH Yee, K Kim, Dynamical mean-field theory within the full-potential methods: Electronic structure of $CeIrIn_5$, $CeCoIn_5$, and $CeRhIn_5$. *Phys. Rev. B* **81**, 195107 (2010).
11. K Haule, Quantum Monte Carlo impurity solver for cluster dynamical mean-field theory and electronic structure calculations with adjustable cluster base. *Phys. Rev. B* **75**, 155113 (2007).
12. P Werner, A Comanac, L de' Medici, M Troyer, AJ Millis, Continuous-Time Solver for Quantum Impurity Models. *Phys. Rev. Lett.* **97**, 076405 (2006).
13. ZP Yin, K Haule, G Kotliar, Spin dynamics and orbital-antiphase pairing symmetry in iron-based superconductors. *Nat. Phys.* **10**, 845–850 (2014).
14. H Mao, et al., Topological states and competing magnetic fluctuations in iron germanides. *Phys. Rev. B* **107**, 115116 (2023).
15. SL Skornyakov, VI Anisimov, Coulomb correlations in a germanide iron-based superconductor: The example of YFe_2Ge_2. *Phys. Rev. B* **107**, 235103 (2023).
16. G Venturini, B Malaman, X-ray single crystal refinements on some RT_2Ge_2 compounds (R = Ca, Y, La, Nd, U; T = Mn-Cu, Ru-Pd): evolution of the chemical bonds. *J. Alloy. Compd.* **235**, 201–209 (1996).
17. J Chen, et al., Unconventional Superconductivity in the Layered Iron Germanide YFe_2Ge_2. *Phys. Rev. Lett.* **116**, 127001 (2016).
18. J Chen, et al., Unconventional Bulk Superconductivity in YFe_2Ge_2 Single Crystals. *Phys. Rev. Lett.* **125**, 237002 (2020).
19. ZP Yin, K Haule, G Kotliar, Kinetic frustration and the nature of the magnetic and paramagnetic states in iron pnictides and iron chalcogenides. *Nat. materials* **10**, 932–5 (2011).
20. N Marzari, AA Mostofi, JR Yates, I Souza, D Vanderbilt, Maximally localized wannier functions: Theory and applications. *Rev. Mod. Phys.* **84**, 1419–1475 (2012).
